# *Salmonella* Effectors SseF and SseG Interact with Mammalian Protein ACBD3 (GCP60) To Anchor *Salmonella*-Containing Vacuoles at the Golgi Network

**DOI:** 10.1128/mBio.00474-16

**Published:** 2016-07-12

**Authors:** Xiu-Jun Yu, Mei Liu, David W. Holden

**Affiliations:** MRC Centre for Molecular Bacteriology and Infection, Imperial College London, London, United Kingdom

## Abstract

Following infection of mammalian cells, *Salmonella enterica* serovar Typhimurium (*S*. Typhimurium) replicates within membrane-bound compartments known as *Salmonella*-containing vacuoles (SCVs). The *Salmonella* pathogenicity island 2 type III secretion system (SPI-2 T3SS) translocates approximately 30 different effectors across the vacuolar membrane. SseF and SseG are two such effectors that are required for SCVs to localize close to the Golgi network in infected epithelial cells. In a yeast two-hybrid assay, SseG and an N-terminal variant of SseF interacted directly with mammalian ACBD3, a multifunctional cytosolic Golgi network-associated protein. Knockdown of ACBD3 by small interfering RNA (siRNA) reduced epithelial cell Golgi network association of wild-type bacteria, phenocopying the effect of null mutations of *sseG* or *sseF*. Binding of SseF to ACBD3 in infected cells required the presence of SseG. A single-amino-acid mutant of SseG and a double-amino-acid mutant of SseF were obtained that did not interact with ACBD3 in *Saccharomyces cerevisiae*. When either of these was produced together with the corresponding wild-type effector by *Salmonella* in infected cells, they enabled SCV-Golgi network association and interacted with ACBD3. However, these properties were lost and bacteria displayed an intracellular replication defect when cells were infected with *Salmonella* carrying both mutant genes. Knockdown of ACBD3 resulted in a replication defect of wild-type bacteria but did not further attenuate the growth defect of a Δ*sseFG* mutant strain. We propose a model in which interaction between SseF and SseG enables both proteins to bind ACBD3, thereby anchoring SCVs at the Golgi network and facilitating bacterial replication.

## IMPORTANCE

Upon invasion of epithelial cells, the majority of vacuoles containing *Salmonella enterica* migrate to the perinuclear region-located Golgi network and remain in this region of the cell during the first few rounds of bacterial replication, forming a clustered microcolony of vacuoles. This process requires the action of SseF and SseG, two effector proteins that are translocated by the *Salmonella* SPI-2 type III secretion system. However, little is known about how they function. Here, we show that both proteins interact with the mammalian Golgi network-associated protein ACBD3. To our knowledge, the SseF-SseG-ACBD3 interaction is the first example of a tethering complex between a pathogen-containing vacuole and a host cell organelle.

## INTRODUCTION

*Salmonella enterica* serovar Typhimurium (*S*. Typhimurium) survives and replicates within a variety of mammalian host cell types, including macrophages and epithelial cells. Uptake of bacteria by host cells results in the formation of the *Salmonella*-containing vacuole (SCV). Acidification and nutritional starvation of the vacuole lumen act as signals for the transcriptional induction of *Salmonella* pathogenicity island 2 (SPI-2) type III secretion system (T3SS) genes (1–4). The type III secretion apparatus assembles in the bacterial cell envelope from approximately 4 to 6 h following bacterial uptake (5, 6). It secretes translocon proteins that are thought to form a pore in the vacuolar membrane. Sensing of host cell cytosolic pH then dissociates a bacterial gatekeeper complex, inducing translocation of over 30 different effector proteins across the vacuolar membrane (5, 7). Effectors translocated by the SPI-2 T3SS localize to the SCV membrane, SCV-associated tubules, and the cell cytoplasm, interfering with normal cell functions and facilitating intracellular bacterial multiplication (7).

SseF and SseG are SPI-2-encoded effectors that localize to SCV membranes and SCV-associated tubules called Sifs that are formed in epithelial cells (8). Both effectors are necessary for the retention of tightly clustered bacterial microcolonies in close proximity to the microtubule-organizing center (MTOC) and Golgi network in epithelial cells (9–14). Intracellular growth and virulence tests showed that single mutant strains lacking SseF or SseG have similar levels of attenuation and that a double mutant lacking both proteins does not have a greater level of attenuation than the single mutants (12). These results indicate that the two proteins are involved in the same virulence function. Indeed, SseF and SseG have been shown to interact following their translocation into host cells (12), suggesting that formation of heterodimers is required to elicit their activity. SseF and SseG share approximately 35% similarity at the amino acid level over their entire lengths and are integral membrane proteins (12, 15). Topological analyses indicate that the C-terminal region of SseF and both N- and C-terminal domains of SseG are exposed to the host cell cytoplasm (9, 11).

Two hypotheses have been proposed to explain the MTOC/Golgi network localization of SCVs in epithelial cells. One is based on evidence showing that microtubule motors influence SCV positioning (10, 11), suggesting that opposing activities of dynein and kinesin-1 occur on the SCV membrane (16, 17). Under normal conditions, dynein activity predominates, leading to a relatively stable association of SCVs with Golgi membranes. The second hypothesis proposes that SCV positioning is controlled by physical tethering of SCV membranes to Golgi network-associated molecules (9, 13). Whatever their precise mechanism(s), SseF and SseG have key functions in regulating SCV localization: imaging of SCVs in live epithelial cells at approximately 8 h postinvasion revealed that Golgi network-associated vacuoles containing wild-type bacteria are relatively immobile, whereas those containing *sseG* mutant bacteria are highly motile and display erratic large-scale movements throughout host cells (13). Close apposition of vacuoles with Golgi membranes could facilitate fusion of Golgi network-derived material with SCVs and thereby provide a source of SCV membrane or proteins, lipids, or other molecules that could be exploited by bacteria for nutrition.

To gain further insights into the functions of SseF and SseG, we carried out a yeast two-hybrid screen and identified ACBD3 as a host protein interacting with SseG. Further genetic analysis, together with infection and biochemical assays, showed that SseF, SseG, and ACBD3 form a trimolecular complex and suggested a model in which an interaction between SseF and SseG is required for both effectors to bind ACBD3.

## RESULTS

### Golgi protein acyl-CoA binding domain containing 3 (ACBD3) interacts with SseG in *Saccharomyces cerevisiae* and is required for SCV-Golgi network association.

In an attempt to identify host cell proteins that interact with SseG, we used pGBT-*sseG* as a bait plasmid in a yeast two-hybrid screen with a cDNA library from HeLa cells. Of 3.6 × 10^7^ transformants, two grew on synthetically defined quadruple dropout (SD-QDO) medium. DNA sequencing revealed that they were identical clones encoding residues 188 to 528 of Golgi complex protein 60/Golgi protein acyl coenzyme A (acyl-CoA) binding domain containing 3 (GCP60/ACBD3) (here referred to as ACBD3).

**FIG 1:**
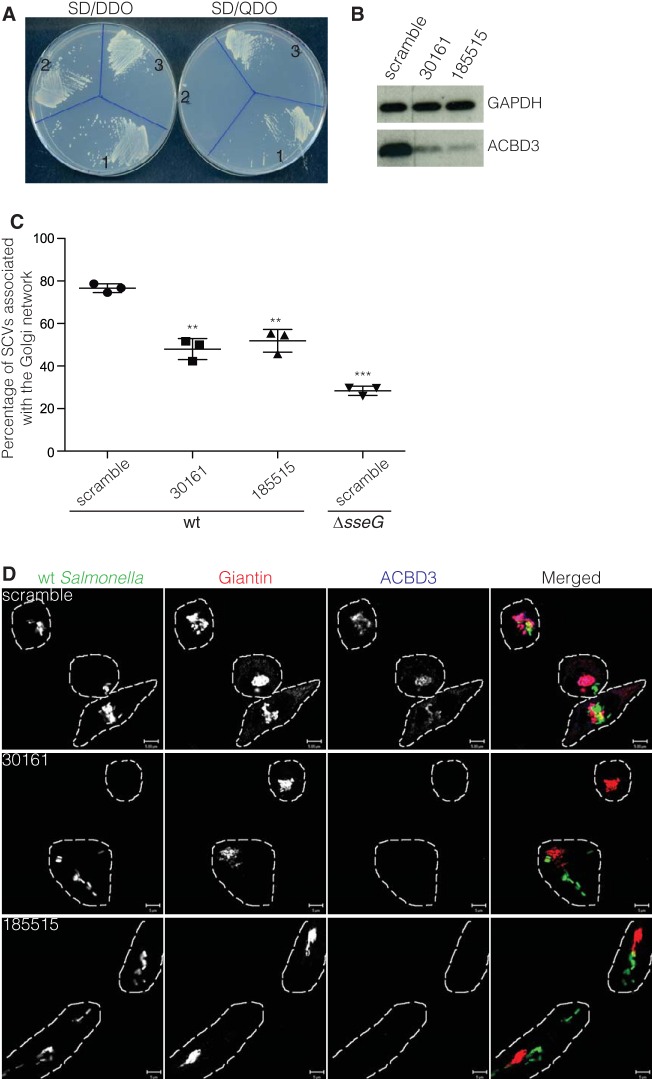
ACBD3 interacts with SseG and is required for the Golgi network association of SCVs. (A) Growth of *S. cerevisiae* PJ69-4A transformants on SD-DDO (SD/-Leu-Trp dropout supplement) and SD-QDO (SD/-Leu-Trp-His-Ade dropout supplement) plates. 1, pGBT-*sseG* + pGAD-cDNA1 (prey plasmid isolated from original yeast two-hybrid screening); 2, pGBT-*sseG* + pGADT7-T (vector used for constructing HeLa cell cDNA library); 3, pGBT-*sseG* + pGAD-*ACBD3_188-528_* (cDNA encoding ACBD3_188-528_ ligated into pGAD424). (B to D) Knockdown of ACBD3 by siRNA affects the Golgi network association of SCVs. HeLa cells were treated with scramble siRNA or ACBD3 siRNAs (oligonucleotide 30161 or 185515) for 72 h and then collected for immunoblotting to check knockdown of ACBD3 (B) or reseeded for infection with *S*. Typhimurium expressing GFP. Fourteen hours after infection, cells were fixed with PFA and labeled for giantin (red) and ACBD3 (blue) for Golgi network association scoring (C) and confocal microscope micrographs (D). (C) Data represent means ± standard errors of the means (SEM) from three independent experiments. The infected cells treated with oligonucleotide 30161 or 185515 were compared with infected cells treated with scramble oligonucleotide. **, *P* < 0.01; ***, *P* < 0.001. (D) Bars, 5 µm. wt, wild type.

The DNA sequence encoding the 188 to 528 residues of ACBD3 was subcloned to pGAD424 to create plasmid pGAD-*ACBD3_188-528_*. This plasmid supported growth of the yeast strain carrying pGBT-*sseG* on SD-QDO (Fig. 1A), indicating that SseG interacts with ACBD3 in yeast. ACBD3 is a conserved and apparently ubiquitously expressed cytosolic Golgi network-associated protein of 60 kDa and is composed of several domains: a long N-terminal acyl-CoA binding domain (ACBD) region, a domain that is predicted to form coiled-coil interactions, and a C-terminal Golgi dynamics (GOLD) domain that interacts with giantin, thereby localizing ACBD3 to Golgi membranes (18).

To investigate the possible involvement of ACBD3 in maintaining the association between SCVs and the Golgi network, endogenous HeLa cell ACBD3 was depleted by small interfering RNA (siRNA) (Fig. 1B and D), and these cells were infected with wild-type *S*. Typhimurium. Knockdown of ACBD3 by two different oligonucleotides resulted in significantly reduced Golgi network association of wild-type bacteria, phenocopying the effect of mutation in *sseG* (Fig. 1C and D). This indicates that ACBD3 is required to maintain the close association between SCVs and the Golgi network.

### SseG^S67G^ does not interact with ACBD3 in yeast but complements the SCV-Golgi network association defect of an *sseG* mutant.

**FIG 2:**
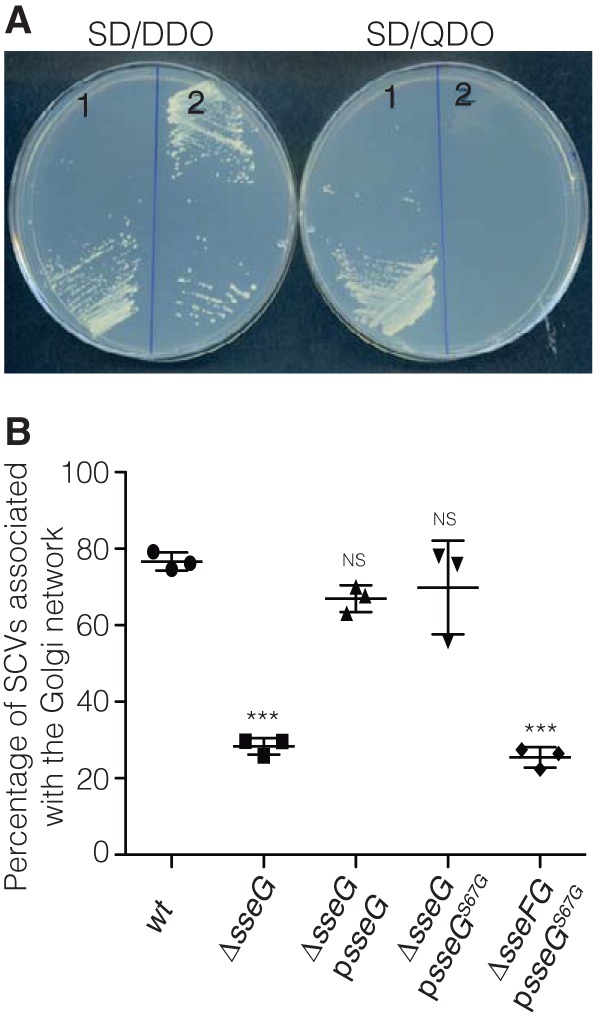
SseG^S67G^ does not interact with ACBD3 but complements the *sseG* mutant for association with the Golgi network. (A) Growth of *S. cerevisiae* PJ69-4A transformants on SD-DDO and SD-QDO plates. 1, pGBT-*sseG* + pGAD-*ACBD3_188-528_*; 2, pGBT-*sseG^S67G^* + pGAD-*ACBD3_188-528_*. (B) SseG^S67G^ complements the *sseG* mutant but not the *sseFG* mutant for association with the Golgi network. HeLa cells were infected for 14 h with indicated strains, fixed, and immunolabeled to detect *Salmonella* and giantin for Golgi network association scoring. Data represent means ± SEMs from three independent experiments. Cells infected with the wild-type (wt) strain were compared with cells infected by other indicated strains. ***, *P* < 0.001; NS, not significant.

As an alternative test of the relevance of the SseG-ACBD3 interaction during *Salmonella* infection, we attempted to obtain a point mutant of SseG that fails to interact with ACBD3 in yeast. To do so, a DNA fragment containing *sseG* flanked by approximately 235 bp of upstream and downstream sequences from pGBT-*sseG* was amplified by error-prone PCR and cotransferred with linearized empty bait vector pGBT9 into the yeast reporter strain bearing pGAD-*ACBD3_188-528_*. Out of 1,032 transformants that were screened, 52 were unable to grow on SD-QDO medium. The recombinant pGBT9 derivatives were isolated from growth-defective yeast strains for DNA sequencing. The majority contained nonsense mutations causing premature stop codons, but a single missense mutation that causes a change of serine 67 to glycine (SseG^S67G^) was found. This *sseG^S67G^* allele was then ligated into pGBT9, and this was used to verify that SseG^S67G^ did not interact with ACBD3 in yeast (Fig. 2A).

To analyze the phenotype of the SseG^S67G^ mutant in infected cells, plasmid p*sseG^S67G^* was constructed and transformed into *S*. Typhimurium carrying a null mutation in *sseG*. The resulting strain was used to infect HeLa cells for 14 h, which were then fixed for immunolabeling of bacteria and giantin, as a marker of the *cis*-Golgi network. There was no significant difference between the level of SCV-Golgi network association in HeLa cells infected with this strain (Δ*sseG* p*sseG^S67G^*) and that in cells infected with an Δ*sseG* mutant carrying the wild-type allele of *sseG* (Δ*sseG* p*sseG*): in both cases, the majority of SCVs were found in close association with the Golgi network (Fig. 2B). However, the presence of p*sseG^S67G^* in a Δ*sseFG* mutant failed to complement the Golgi network association defect of the double mutant strain (Fig. 2B), indicating that SseG^S67G^ requires SseF for its function. These results show that lack of interaction between SseG^S67G^ and ACBD3 in yeast does not prevent SCVs from associating with the Golgi network. This suggests that ACBD3 might not be a physiologically relevant host cell binding partner of SseG. Alternatively, since SseG interacts with SseF and the Golgi network association phenotype of *S*. Typhimurium also requires SseF (12), it is possible that ACBD3 also interacts with SseF (or other effectors) in the presence of SseG^S67G^, to maintain SCV-Golgi network association.

### SseF_1-166_ interacts with ACBD3 in yeast, and simultaneous disruption of SseF-ACBD3 and SseG-ACBD3 interactions results in reduced association of SCVs with the Golgi network.

**FIG 3:**
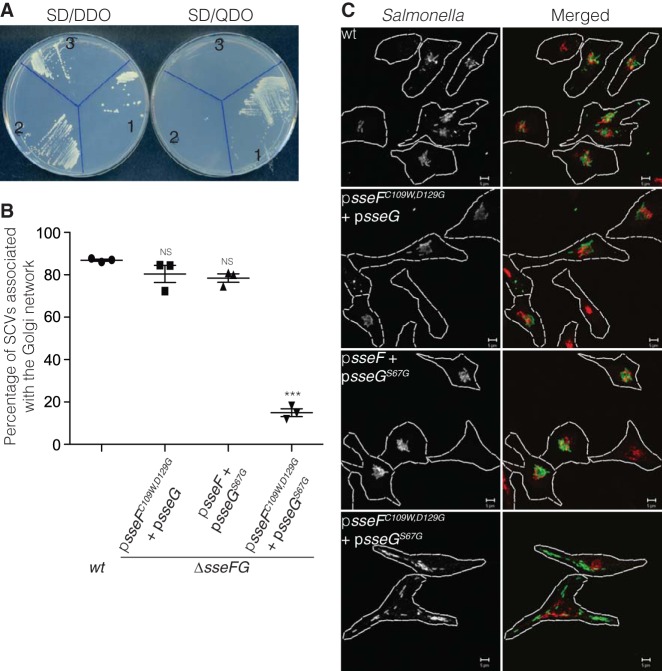
SseF_1-166_ but not SseF_1-166_^C109W,D129G^ interacts with ACBD3, and the *sseFG* mutant strain expressing SseF^C109W,D129G^ and SseG^S67G^ does not associate with the Golgi network. (A) Growth of *S. cerevisiae* PJ69-4A transformants on SD-DDO and SD-QDO plates. 1, pGBT-*sseF_1-166_* + pGAD-*ACBD3_188-528_*; 2, pGBT-*sseF_105-260_* + pGAD-*ACBD3_188-528_*; 3, pGBT-*sseF_1-166_^C109W,D129G^* + pGAD-*ACBD3_188-528_*. (B) Golgi network association of wild type (wt) or *sseFG* mutant carrying the indicated plasmids. HeLa cells were infected for 14 h with indicated strains, fixed, and immunolabeled to detect *Salmonella* and giantin. Data represent means ± SEMs from three independent experiments. Cells infected with the wild-type strain were used for comparison with cells infected by other indicated strains. ***, *P* < 0.001; NS, not significant. (C) Representative micrographs taken from the experiment in panel B. Green, *Salmonella*; red, giantin. Bars, 5 µm.

Next, we determined if SseF interacts with ACBD3 in yeast. As full-length SseF (amino acids 1 to 260) fused to the GAL4 DNA-binding domain (GBD::SseF) was toxic to yeast cells (results not shown), plasmids pGBT-*sseF*_1-166_ and pGBT-*sseF*_105-260_ were constructed and the encoded truncated proteins were tested for their ability to bind ACBD3. The yeast reporter strain carrying pGAD-*ACBD3_188-528_* grew on SD-QDO medium when transformed with pGBT-*sseF*_1-166_ but not with pGBT-*sseF*_105-260_ (Fig. 3A), indicating that the N-terminal 166 amino acids of SseF are sufficient to interact with ACBD3 in this assay.

To isolate a mutation of SseF_1-166_ that prevents interaction with ACBD3, a screen was carried out using variants of SseF_1-166_ generated by error-prone PCR in yeast cells containing pGAD-*ACBD3_188-528_*, as described above for *sseG*. Out of 1,670 transformants that were screened, 58 were unable to grow on SD-QDO medium. Of these, the minimally perturbed mutant that failed to support growth of the yeast reporter strain carried two amino acid substitutions (SseF_1-166_^C109W,D129G^) (Fig. 3A). To test the effect of the double substitution mutant on SCV-Golgi network association, plasmid p*sseF^C109W,D129G^* was constructed (encoding full-length SseF with two substitutions) and transformed into an *sseFG* double mutant carrying p*sseG*. HeLa cells were infected with the Δ*sseFG* p*sseF^C109W,D129G^* p*sseG* strain for 14 h and then fixed for immunolabeling of *Salmonella* and the Golgi network. There was no significant difference between the level of SCV-Golgi network association in HeLa cells infected with this strain and that in cells infected with the wild-type strain or with the double mutant strain carrying the wild-type *sseF* allele and p*sseG^S67G^*. In each case, the majority of SCVs were found in close association with the Golgi network (Fig. 3B and C), indicating that, as for the SseG single substitution, disrupting the SseF-ACBD3 interaction in yeast does not prevent SCVs from associating with the Golgi network.

It remained possible that, in infected HeLa cells, both SseF and SseG interact with ACBD3 and that the wild-type form of one *Salmonella* protein is sufficient to mediate SCV-Golgi network association in the presence of the single or double point mutant version of the other (i.e., in Δ*sseFG* p*sseF^C109W,D129G^* + p*sseG* strain*-*infected HeLa cells, SseG interacts with ACBD3 to mediate Golgi network association, and in Δ*sseG* p*sseG^S67G^* strain-infected HeLa cells, SseF interacts with ACBD3 to mediate Golgi network association). Therefore, to test if simultaneous disruption of the SseG-ACBD3 and SseF-ACBD3 interactions that occur in yeast impairs SCV-Golgi network association, p*sseG^S67G^* and p*sseF^C109W,D129G^* were cotransformed into the Δ*sseFG* double null mutant, and this strain was used to infect HeLa cells. In contrast to the expression of the point mutants of either SseF or SseG, this strain was unable to complement the Golgi network association defect of the Δ*sseFG* mutant (Fig. 3B and C). Therefore, these results show that, when expressed from *Salmonella,* the single and double point mutant versions of SseG and SseF are functional in the presence of the wild type but not the point mutant version of the other protein. Since these point mutants have lost their ability to interact individually with ACBD3 in yeast, the results suggest that in infected cells, SseF and SseG might form a complex with ACBD3 that is required to maintain the association of SCVs with the Golgi network.

### SseF and SseG interact with ACBD3 in infected HeLa cells.

**FIG 4:**
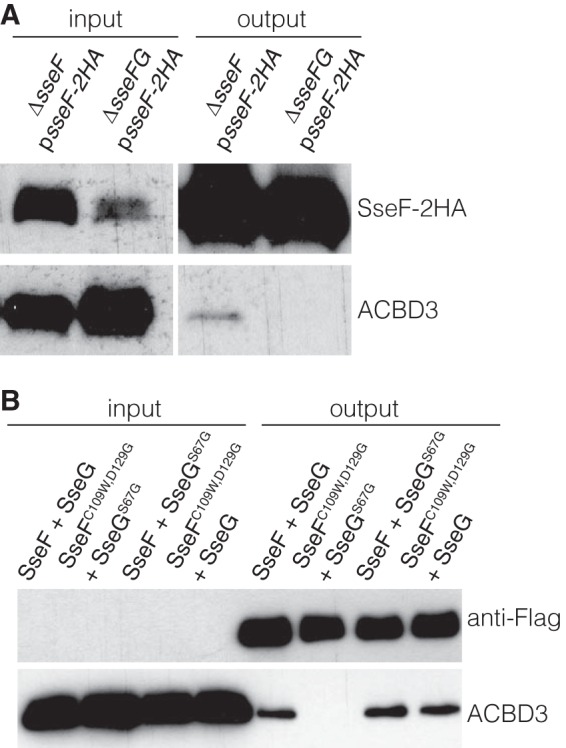
Coimmunoprecipitation assays. (A) SseG is required for SseF to interact with ACBD3 in infected HeLa cells. HeLa cells were infected with indicated bacterial strains for 14 h and then lysed to immunoprecipitate SseF-2HA. Samples were separated by SDS-PAGE for immunoblotting. (B) SseF^C109W,D129G^ and SseG^S67G^ fail to interact with ACBD3. HeLa cells were infected with the *sseFG* mutant expressing Flag-tagged SseG or SseG^S67G^ and untagged SseF or SseF^C109W,D129G^ for 14 h. Cell lysates were prepared to immunoprecipitate SseG-Flag or SseG^S67G^-Flag. Samples were separated by SDS-PAGE for immunoblotting.

To determine if interaction between ACBD3 and SseF occurs in infected cells, HeLa cells were infected for 14 h with either Δ*sseF* or Δ*sseFG* mutant strains expressing epitope-tagged SseF-2HA from p*sseF-2HA*. Cells were lysed and subjected to immunoprecipitation with antihemagglutinin (anti-HA) antibody-conjugated beads. In Δ*sseF* p*sseF-2HA* strain-infected cells (in which the majority of SCVs were Golgi network associated), ACBD3 was reproducibly coimmunoprecipitated with SseF-2HA (Fig. 4A). However, ACBD3 was not coimmunoprecipitated by SseF-2HA in cells infected with the Δ*sseFG* p*sseF-2HA* strain (Fig. 4A). This demonstrates that SseF interacts with ACBD3 in infected HeLa cells and that this interaction depends on the presence of SseG.

Next, we tested the ability of translocated SseG^S67G^ and SseF^C109W,D129G^ to interact with ACBD3 in epithelial cells. For this, we constructed plasmids p*sseF sseG-Flag*, p*sseF^C109W,D129G^ sseG^S67G^*-Flag, p*sseF sseG^S67G^*-Flag, and p*sseF^C109W,D129G^* sseG-Flag, in which expression of SseF and SseG or their variants is under the control of their endogenous *sseA* promoter. Plasmids were transformed into the Δ*sseFG* mutant, and the resulting strains were used to infect HeLa cells for 14 h. Cell lysates were incubated with anti-Flag antibody-conjugated beads to immunoprecipitate Flag-tagged SseG or SseG^S67G^. ACBD3 was reproducibly coimmunoprecipitated with SseG-Flag or SseG^S67G^-Flag from cells infected with Δ*sseFG* p*sseFsseG-Flag*, Δ*sseFG* p*sseFsseG^S67G^*-Flag, or Δ*sseFG* p*sseF^C109W,D129G^* sseG-Flag strains (Fig. 4B). However, ACBD3 was not coimmunoprecipitated with SseG^S67G^-Flag from cells infected with the Δ*sseFG* p*sseF^C109W,D129G^ sseG^S67G^*-Flag strain, despite efficient immunoprecipitation of SseG^S67G^-Flag. These results demonstrate that the ability of SseG to interact with ACBD3 in HeLa cells is prevented only by concurrent expression of SseF and SseG mutants that do not interact with ACBD3 in yeast. The results are also consistent with the conditional functionality of the single and double point mutant versions of SseG and SseF with respect to Golgi network localization in infected HeLa cells (Fig. 3B). Taken together with the effect of depletion of ACBD3 on Golgi network localization of SCVs, our results indicate that an interaction between SseF, SseG, and ACBD3 is required to maintain the association between SCVs and the epithelial cell Golgi network.

### Interactions with ACBD3 facilitate intracellular bacterial replication.

**FIG 5:**
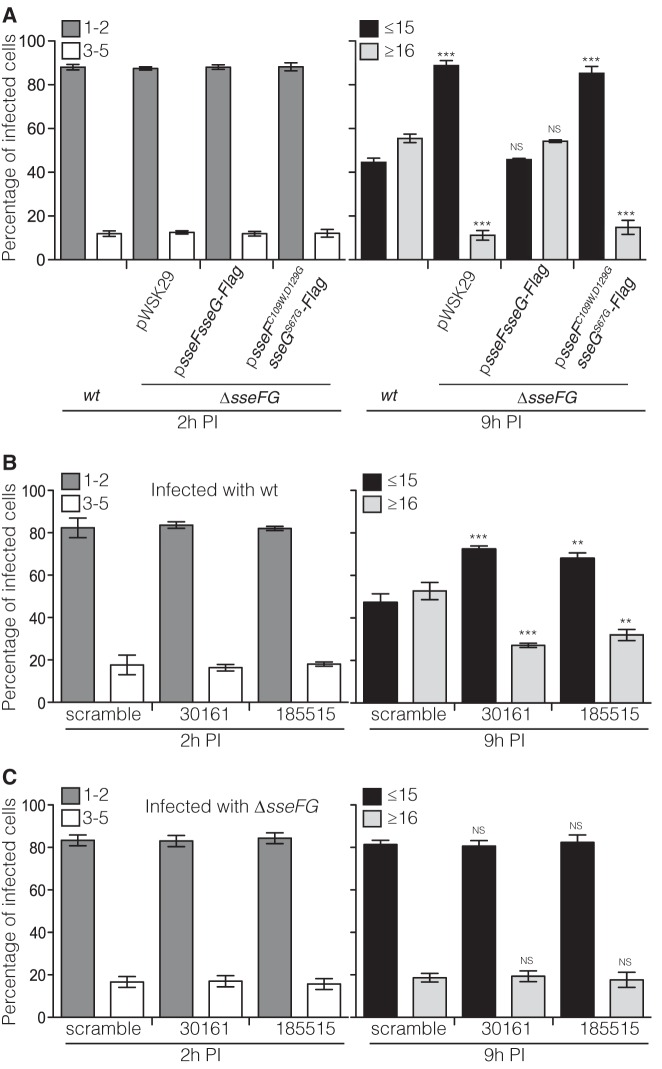
Interaction of ACBD3 with SseF and SseG facilitates *Salmonella* replication in HeLa cells. (A) SseF^C109W,D129G^ and SseG^S67G^ fail to rescue the *sseFG* mutant for replication in HeLa cells. HeLa cells were infected with indicated bacterial strains for 2 h or 9 h and then fixed with PFA and labeled for *Salmonella* before the number of bacteria in infected cells was quantified by microscopy. Cells infected with the *sseFG* mutant or with the *sseFG* mutant carrying the indicated plasmids were compared with cells infected with the wild-type (wt) strain at corresponding time points. Data represent means ± SEMs from three independent experiments. ***, *P* < 0.001; NS, not significant. (B) Knockdown of ACBD3 impairs replication of wild-type *Salmonella* in HeLa cells. HeLa cells were treated with scramble siRNA or ACBD3 siRNAs (oligonucleotide 30161 or 185515) for 72 h and reseeded for infection with wild-type *S*. Typhimurium expressing GFP. At 2 h or 9 h after infection, cells were fixed with PFA and the numbers of bacteria in infected cells were quantified by microscopy. The oligonucleotide 30161- or 185515-treated cells were compared with scramble oligonucleotide-treated cells at corresponding time points. Data represent means ± SEMs from three independent experiments. **, *P* < 0.01; ***, *P* < 0.001. (C) Knockdown of ACBD3 does not further attenuate the replication defect of the *sseFG* mutant in HeLa cells. HeLa cells were treated with scramble siRNA or ACBD3 siRNAs (oligonucleotide 30161 or 185515) for 72 h and reseeded for infection with the *sseFG* mutant expressing GFP. At 2 h or 9 h after infection, cells were fixed with PFA and the numbers of bacteria in infected cells were quantified by microscopy. The oligonucleotide 30161- or 185515-treated cells were compared with scramble oligonucleotide-treated cells at corresponding time points. Data represent means ± SEMs from three independent experiments. NS, not significant. PI, postinvasion.

To analyze the importance of interactions between SseF, SseG, and ACBD3 in *Salmonella* replication in epithelial cells, HeLa cells were infected with different *Salmonella* strains for 2 h or 9 h and then fixed and labeled with anti-*Salmonella* antibody to enumerate intracellular bacteria by microscopy. At 2 h postuptake, intracellular bacterial numbers were similar in cells infected with different strains (Fig. 5A). At 9 h postuptake, approximately 54% of cells infected with the Δ*sseFG* p*sseFsseG-Flag* strain had more than 16 bacteria, a level which was similar to that of wild-type-infected cells. However, only approximately 14% of cells infected with the Δ*sseFG* p*sseF*^C109W,*D129G*^ *sseG^S67G^*-Flag strain had more than 16 bacteria, similar to the Δ*sseFG* pWSK29 strain-infected cells (Fig. 5A). This demonstrates that when combined, the presence of single and double point mutants of *sseG* and *sseF* causes a replication defect in infected cells. Since these mutants also disrupt the interaction with ACBD3 (Fig. 4B), these data provide correlative evidence that interactions between SseF, SseG, and ACBD3 are required for bacterial replication in epithelial cells.

Next, we investigated the effect of depletion of ACBD3 on bacterial growth in HeLa cells. ACBD3 was depleted from HeLa cells using one of two different siRNA oligonucleotides prior to infection with wild-type green fluorescent protein (GFP)-expressing *S*. Typhimurium for 2 h or 9 h. Cells were fixed and examined by microscopy, as described above. At 2 h postuptake, there were 1 or 2 bacteria in approximately 80% of infected cells pretreated with scramble siRNA or ACBD3 siRNA. By 9 h postuptake, approximately 53% of infected cells pretreated with scramble siRNA had more than 16 bacteria, but approximately 30% of infected cells pretreated with either of the two ACBD3-targeting siRNAs had more than 16 bacteria (Fig. 5B). This demonstrates that depletion of ACBD3 inhibits bacterial replication.

To determine if the growth advantage conferred by SseF and SseG is dependent on ACBD3, the intracellular growth of the Δ*sseFG* double mutant strain was measured following depletion of ACBD3. HeLa cells were pretreated with scramble or ACBD3-specific siRNAs and then infected with the Δ*sseFG* double mutant strain expressing GFP. At 2 h postinvasion, there was no detectable difference between numbers of Δ*sseFG* mutant bacteria in scramble- or ACBD3-depleted cells (Fig. 5C). At 9 h postinvasion, the numbers of Δ*sseFG* mutant bacteria in ACBD3-depleted cells were indistinguishable from those of the same mutant in ACBD3-replete cells (Fig. 5C). Therefore, loss of SseF, SseG, or ACBD3 all leads to an intracellular growth defect, but absence of ACBD3 does not further attenuate the growth defect of the Δ*sseFG* mutant strain. This provides direct evidence that the replicative capacity mediated by SseF and SseG requires the presence of ACBD3.

## DISCUSSION

Upon invasion of epithelial cells, the majority of SCVs migrate to the perinuclear region-located MTOC and Golgi network and remain in this region of the cell during the first few rounds of bacterial replication, forming a clustered microcolony of SCVs. Bacterial strains carrying mutations in *sseF* or *sseG* move to the MTOC/Golgi network with kinetics similar to those of wild-type bacteria but fail to form stable microcolonies. Instead, their SCVs move erratically throughout the cytoplasm of infected cells, resulting in a scattered phenotype (9, 12, 13). This phenotype suggests that SseF and SseG function by tethering SCVs to a Golgi network-associated compartment and/or by modulating the activities of microtubule motors, which are known to interact with SCVs (10, 11, 14, 19, 20), in a way that favors minus-end-directed dynein activity on SCVs.

Within SPI-2, *sseF* and *sseG* are located in the same operon as genes encoding translocon components of the T3SS (21) and, unlike several effectors, appear to be functional in all serovars of *S. enterica* (22). This suggests that they were acquired early in the development of the effector repertoire of this T3SS and that their functions are important for bacterial virulence in different mammalian hosts. In this study, we have gained more insight into SseF and SseG and how they might function to localize SCVs at the Golgi network. A yeast two-hybrid screen with SseG and cDNAs from HeLa cells led to the identification of ACBD3 as an SseG-interacting protein. That ACDB3 might be a bona fide binding partner was suggested by its cytoplasmic Golgi network-associated location, interaction with the Golgi network-tethering protein giantin (18), and the scattered distribution of SCVs containing wild-type bacteria in epithelial cells that had been depleted of ACBD3. However, in view of the high rate of false-positive hits that can result from screens of this type, we sought additional evidence for the physiological significance of SseG-ACBD3 interactions by assessing the functionality of a minimally altered SseG mutant that failed to interact with ACBD3 in yeast. A single-amino-acid variant that did not interact with ACBD3 in yeast was obtained, but this was functional in terms of its ability to rescue the Golgi network association defect of an *S*. Typhimurium *sseG* null mutant. Yeast two-hybrid assays also established ACBD3 as a potential protein interacting with SseF, and in this case, a double-amino-acid variant of SseF that lost the interaction with ACBD3 in yeast also retained the ability to complement an *S*. Typhimurium *sseF* null mutant for Golgi network localization. However, when the mutant versions of SseF and SseG were combined in the same bacterial strain, these failed to complement the Golgi network association and intracellular growth defects of the double null mutant. The ability of SseG to immunoprecipitate ACBD3 in infected cells was also lost if both SseF and SseG were present in their mutant forms, but SseG-ACBD3 binding was retained if either the SseF or SseG variant was coexpressed with the corresponding wild-type protein. Furthermore, knockdown of ACBD3 reduced growth of intracellular wild-type *Salmonella* but did not exacerbate the growth defect of the Δ*sseFG* mutant strain. Together, these experiments provide strong evidence that the functionality of point mutant forms of SseF or SseG is dependent on a wild-type version of the corresponding protein and that they interact with ACBD3 to mediate Golgi network association of SCVs and bacterial replication in epithelial cells.

**FIG 6:**
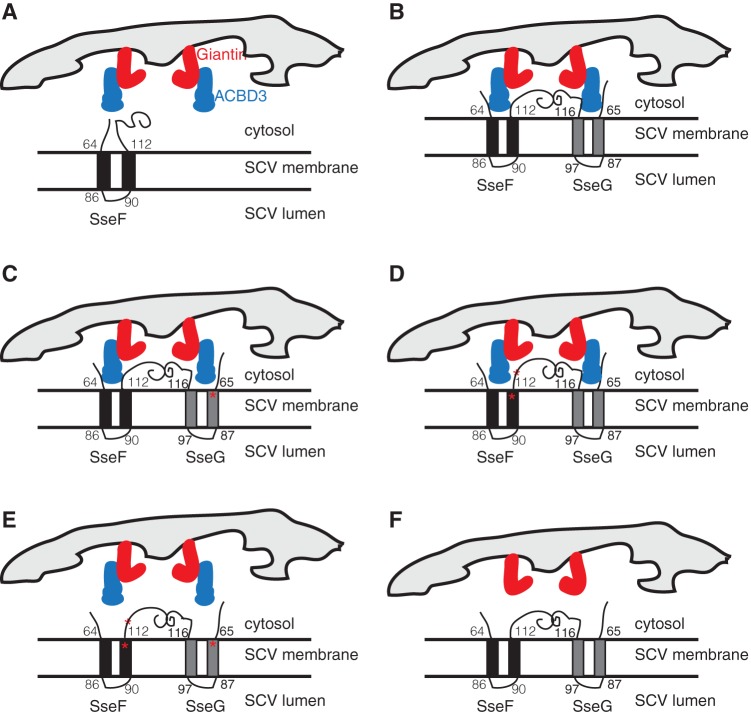
Model of interaction between ACBD3 and SseF and SseG. (A) ACBD3 (blue) interacts with the integral membrane protein giantin (red) at the Golgi network. In the absence of SseG, SseF assumes a closed conformation and is unable to interact with ACBD3. (B) When both SseG and SseF are present, they interact. This changes their conformations to allow them to interact with ACBD3. (C) A point mutant of SseG (SseG^S67G^) is unable to interact with ACBD3 but remains functional by enabling SseF to interact with ACBD3. (D) A double mutant of SseF (SseF^C109W,D129G^) is unable to interact with ACBD3 but remains functional by enabling SseG to interact with ACBD3. (E) Simultaneous disruption of SseF-ACBD3 and SseG-ACBD3 interactions by coexpression of mutant forms of both SseF and SseG prevents SCVs from stable association with the Golgi network. (F) Depletion of ACBD3 prevents SCVs from associating with the Golgi network.

Both SseF and SseG are integral membrane proteins that form heterodimers (12, 15). Topological experiments have revealed that the N and C termini of both proteins are exposed to the cytoplasm (9, 11) (see also Fig. S1 in the supplemental material). The evidence presented here is consistent with a model (Fig. 6) in which the S67G point mutation in SseG localizes within or at the cytoplasmic interface of the first transmembrane region. In SseF, one mutation (C109W) is located near the cytoplasmic face of the second transmembrane region, and the other (D129G) lies in the C-terminal region exposed to the cytoplasm. In cells infected with strains expressing either SseF or SseG, we propose that the bacterial effector is held in a closed or autoinhibited conformation that does not allow its interaction with ACBD3 (Fig. 6A). In the yeast two-hybrid assay, it is possible that fusion of the GAL4 DNA-binding domain to SseG disrupts its autoinhibited state, enabling it to bind ACBD3. In our model, the point mutations affect the correct folding of the cytoplasmic regions and disrupt the binding interfaces between effectors and ACBD3. Simultaneous production of both wild-type effectors by intracellular bacteria leads to their heterodimerization, possibly via their C-terminal domains, and this exposes the regions of SseF and SseG that interact with ACBD3 (Fig. 6B). Thus, although SseF or SseG point mutants fail to bind ACBD3 either in yeast or in infected cells, if the corresponding protein is present in its wild-type form, the formation of a heterodimer exposes a functional ACDB3 binding region, which would account for the conditional phenotype of the point mutants (Fig. 6C and D). Production of SseF and SseG mutants from the same bacterial cell does not allow either protein to interact with ACBD3, and this, or the absence of ACBD3, causes a loss of tethering and Golgi network association (Fig. 6E and F). Attempts to test this model experimentally have been hampered by the instability of the truncated forms of both proteins (results not shown). A detailed mechanistic understanding of the interactions between SseF, SseG, and ACBD3 now requires knowledge of the three-dimensional structures of the effectors in isolation, as heterodimers, and when bound to ACBD3.

Correct positioning of eukaryotic organelles is essential for the normal processes of cell function and division. Several tethering complexes that ensure stable associations and close proximity of opposing organelle membranes have been identified. These include complexes connecting mitochondria and the endoplasmic reticulum (ER), peroxisomes, and autophagosomes, as well as complexes between the ER and plasma membrane. These interactions often involve high-affinity lipid binding domains and integral membrane proteins (23) that form a physical bridge between the two organelles. The contact sites do not usually fuse but facilitate interorganelle signaling, ion transport, and nonvesicular lipid exchange across the two membranes (24, 25). To our knowledge, the SseF-SseG-ACBD3 interaction is the first example of a tethering complex between a pathogen-containing vacuole and host cell organelles. Stable and close apposition between SCV and Golgi membranes might enable *Salmonella* to recruit Golgi network-derived proteins or lipids such as cholesterol, some of which is transported through the Golgi network and accumulates in the SCV membrane (26, 27). Another possibility is that phosphoinositide 4-phosphate [PI(4)P] present in the SCV membrane (28) is generated by the activity of phosphatidylinositol 4-kinase IIIβ, a binding partner of ACBD3 (29). However, using the PI(4)P-binding domain of *Legionella pneumophila* SidC as a probe to visualize SCV-associated PI(4)P (28), we were unable to detect obvious differences in labeling between SCVs containing wild-type and Δ*sseFG* mutant bacteria (results not shown). Alternatively, the significance of the interaction between SseF, SseG, and ACBD3 might be indirect, by retaining SCVs at a cellular location where they are more likely to encounter Golgi network-derived or endosomal tubules or vesicles with which they could fuse. Whatever the precise consequences of the SseF-SseG-ACBD3 interaction, our growth assays (Fig. 5) indicate that it is important for efficient replication of vacuolated *Salmonella* within epithelial cells.

## MATERIALS AND METHODS

### Bacterial strains and growth conditions.

*Salmonella enterica* serovar Typhimurium wild-type strain NCTC 12023 and its isogenic mutant derivative Δ*sseF*::*aphT* (Δ*sseF*) and Δ*sseG*::*aphT* (Δ*sseG*) (21) and Δ*sseFG* (12) strains were used in this study. Bacteria were grown in Luria-Bertani (LB) medium supplemented with ampicillin (50 µg ml^−1^) and/or chloramphenicol (34 µg ml^−1^) for plasmid-containing strains as appropriate.

### Construction of plasmids.

Primers (sequences of primers used in this study are listed in Table S1 in the supplemental material) sseGyf and sseGyr, sseFyf and sseF_166_yr, or sseF105yf and sseFyr were used to amplify *sseG*, *sseF_1-166_*, and *sseF_105-260_* by PCR from *S. enterica* serovar Typhimurium strain 12023 genomic DNA. The PCR products were digested with EcoRI and XhoI and ligated into the EcoRI and SalI sites of pGBT9 to create pGBT-*sseG*, pGBT-*sseF_1-166_*, and pGBT-*sseF_105-260_* for the yeast two-hybrid assay. Plasmid pGAD-cDNA1 (see below) was used as the template to amplify DNA sequence encoding ACBD3_188-528_ with primers ACBD3_188_f and ACBD3_528_r, and the PCR product was digested with EcoRI and XhoI and ligated into the EcoRI and SalI sites of pGAD424 to construct pGAD-*ACBD3_188-528_*.

Primers sseGS67Gf and sseGS67Gr were used for reverse PCR by using p*sseG* (21) as the template to introduce the S67G point mutation of *sseG* into complementing plasmid p*sseG^S67G^*. To create p*sseF^C109W,D129G^*, overlapping PCR was carried out. In the first round of PCR, primers sseF1 and sseF2 were used to amplify *sseF_1-166_^C109W,D129G^* from pGBT-*sseF_1-166_^C109W,D129G^*, and primers sseF3 and sseF4 were used for *sseF_161-260_* from *Salmonella* genomic DNA. In the second round of PCR, the mixture of purified PCR products *sseF_1-166_^C109W,D129G^* and *sseF_161-260_* was used as the template to amplify *sseF^C109W,D129G^* by using primers sseF1 and sseF4. The resulting PCR product was digested with EcoRV and SacI and used to replace *sseF-2HA* of p*sseF-2HA* (5) to create p*sseF^C109W,D129G^*.

To coexpress *sseF* and *sseG-Flag* or their variants under the control of their native *sseA* promoter from a low-copy-number plasmid, pWSK29 (30), the corresponding DNA fragments were amplified by PCR as the following: primers sseF1 and sseF5 for *sseF* by using strain 12023 genomic DNA as the template and *sseF^C109W,D129G^* by using plasmid p*sseF^C109W,D129G^* as the template and primers sseG1 and sseGflagr for *sseG* by using 12023 genomic DNA as the template and *sseG^S67G^* by using plasmid p*sseG^S67G^* as the template. DNA fragments of *sseF* or *sseF^C109W,D129G^* were digested with EcoRV and BamHI, and fragments of *sseG* or *sseG^S67G^* were digested with BamHI and SacI. Plasmid p*sseF-2HA* was digested with EcoRV and SacI to remove insert *sseF-2HA*, and a pWSK29-*sseA* promoter fragment recovered from an agarose gel was used for ligation with digested *sseF* or *sseF^C109W,D129G^* and *sseG* or *sseG^S67G^* to create p*sseF sseG-Flag*, p*sseF^C109W,D129G^ sseG^S67G^*-Flag, p*sseF sseG^S67G^*-Flag, and p*sseF^C109W,D129G^* sseG-Flag.

All the plasmids constructed in this study were verified by DNA sequencing.

### Yeast two-hybrid assay.

pGBT-*sseG* was used as a bait plasmid to screen a cDNA library from HeLa cells (pretransformed human HeLa Matchmaker cDNA library; DB Biosciences; catalog no. HY4027AH) according to the manufacturer’s instructions. Of 3.6 × 10^7^ transformants, two grew on SD-QDO stringent selective medium (SD/-Leu-Trp-His-Ade dropout supplement). Plasmids were prepared from the yeast strain and transformed into *Escherichia coli* KC8, and transformation mixtures were plated onto M9 medium with leucine dropout supplement to select and isolate the prey plasmid (named pGAD-cDNA1 and -2) for DNA sequencing.

For the random mutagenesis screens, primers GBT641 and GBT1134r were used to amplify *sseG* or *sseF_1-166_* and its 235-bp flanking fragments on vector from bait plasmid pGBT-*sseG* or pGBT-*sseF_1-166_* by using the GeneMorph random mutagenesis kit (Stratagene; catalog no. 600550). The PCR product and empty bait vector pGBT9 linearized with EcoRI and PstI were cotransferred into the *Saccharomyces cerevisiae* PJ69-4A strain carrying prey plasmid pGAD-*ACBD3_188-528_* and plated onto SD-DDO (SD/-Leu-Trp dropout supplement) plates to select transformants. The transformants were then tested for their inability to grow on SD-QDO medium. Plasmids from transformants failing to grow on SD-QDO medium were isolated and transformed into *E. coli* KC8, and the transformation mixture was plated onto M9 plates supplied with tryptophan dropout supplement to select and isolate the bait plasmid for DNA sequencing. Only a missense mutation(s) in *sseG* or *sseF_1-166_* was subcloned onto pGBT9 to verify its inability to interact with ACBD3 in a yeast two-hybrid assay.

### Antibodies.

The following primary antibodies were used for immunofluorescence labeling or immunoblotting: mouse anti-ACBD3 (Sigma) at a 1:1,000 dilution for immunofluorescence labeling and a 1:3,000 dilution for immunoblotting; mouse anti-glyceraldehyde-3-phosphate dehydrogenase (anti-GAPDH) (Abcam) at a 1:1,000 dilution and mouse anti-Flag (M2; Sigma) at a 1:2,000 dilution for immunoblotting; rabbit antigiantin (Covance) at a 1:1,000 dilution, rabbit anti-TGN46 (LifeSpan Biosciences) at a 1:500 dilution, and goat anti-*Salmonella* antibody (CSA-1; Kirkegaard & Perry Laboratories) at a 1:200 dilution for immunofluorescence labeling; and mouse anti-HA (HA.11; Covance) at a 1:5,000 dilution for immunoblotting and a 1:1,000 dilution for immunofluorescence labeling.

Rhodamine Red X-conjugated donkey anti-mouse or anti-rabbit antibody, donkey anti-goat or donkey anti-rabbit–cyanine 2 (Cy2) antibody, and Cy5-conjugated donkey anti-mouse antibody were purchased from Jackson ImmunoResearch Laboratories, Inc. (West Grove, PA), for immunofluorescence labeling. Horseradish peroxidase (HRP)-conjugated goat anti-rabbit and goat anti-mouse antibodies were purchased from Dako for immunoblotting.

### Cell culture, siRNA transfection, and bacterial infection.

The HeLa (clone HtTA1) cell line was obtained from the European Collection of Cell Cultures. Cells were maintained in Dulbecco’s modified Eagle’s medium (DMEM) supplemented with 10% fetal bovine serum (Sigma) at 37°C in a humidified atmosphere containing 5% (vol/vol) CO_2_. HeLa cells were treated twice with duplex RNA oligonucleotides to knock down ACBD3 as described previously (31). ACBD3 was targeted with the oligonucleotides 30161 (GGUUGGAUUCUUUGAUGUGTT) or 185515 (AUUGAUGGCUGCCAGCAUACACCUC). The scrambled sequence (AAACUUGUCGACGAGAAGCAAUU) was used as a control.

For bacterial infection, HeLa cells were seeded at a density of approximately 5 × 10^4^ cells per well in 24-well microtiter plates 16 to 20 h before infection. Infection with *S*. Typhimurium was done as described previously (32).

### Immunofluorescence microscopy.

Cells were fixed, permeabilized, and incubated with antibodies as described previously (32). Labeled cells were analyzed by using a fluorescence microscope (BX50; Olympus) or a laser scanning confocal microscope (LSM510; Carl Zeiss). Quantification of the number of bacteria associated with the Golgi network was carried out as described previously (9). At least 100 infected cells were scored blind in each experiment. The images were processed using Adobe Photoshop.

A simplified scoring system, in which bacterial numbers were binned into two groups at 2 h postuptake (1 to 2 bacteria/cell or 3 to 5 bacteria/cell) and 9 h postuptake (≤15 bacteria/cell or ≥16 bacteria/cell), was used to measure intracellular bacterial growth by microscopy. Assessing bacterial replication in this way is more tedious than counting bacterial CFU following host cell lysis but can provide a more accurate estimation of net intracellular growth if, for example, the bacterial strains induce different levels of host cell lysis.

### Coimmunoprecipitation.

HeLa cells grown in 150-mm dishes (Corning, United Kingdom) were infected with *S*. Typhimurium for 14 h. Phosphate-buffered saline (PBS)-washed cells were scraped with a rubber policeman, centrifuged for 5 min at 100 × *g,* and resuspended in 500 µl of lysis buffer (PBS–5% glycerol–0.5% Triton X-100–1 mM phenylmethylsulfonyl fluoride [PMSF]) for 30 min at 4°C. Lysates were centrifuged for 10 min at 16,000 × *g* to remove debris before being precleaned with 20 µl of protein G agarose for 1 h (Pierce). The precleaned lysate was mixed with 40 µl of anti-HA antibody-conjugated agarose (Pierce) or anti-Flag antibody-conjugated M2 agarose (Sigma) and incubated at 4°C for 2 h to immunoprecipitate HA-tagged SseF or Flag-tagged SseG. The immunoprecipitates were washed 4 times with lysis buffer and then eluted with 50 µl of 2 mg/ml HA peptide or 0.1 mg/ml Flag peptide. Sample proteins were separated by SDS-PAGE and analyzed by immunoblotting with appropriate antibodies.

### Topological analysis of SseF.

HeLa cells were transfected with 0.2 µg/ml plasmid pCMVHA-*sseF* (12) for 16 h by using the jetPEI reagent (Qbiogene), as detailed by the manufacturer. Cells were permeabilized with 25 µg/ml digitonin (Sigma) for 5 min on ice (33) and then fixed with 3% paraformaldehyde (PFA) before immunolabeling. The fixed cells were labeled with antibodies in the absence of detergent or in the presence of 0.1% Triton X-100 as a positive control.

### Statistical analysis.

All graphs show the mean ± the standard error of the mean (SEM) for three independent experiments. Statistical analyses were performed with Prism 5 software (GraphPad) using one-way analysis of variance (ANOVA) and Dunnett *post hoc* analyses. Probability (*P*) values of 0.05 or less were considered significant.

## SUPPLEMENTAL MATERIAL

Figure S1  Alignment and topology of SseF and SseG. (A) Alignment of amino acid sequences of SseF and SseG. The alignment was generated by ClustalW (http://www2.ebi.ac.uk/clustalw/). The black arrow indicates the point mutation of SseG; gray arrows indicate the point mutations of SseF. The putative transmembrane regions predicted with TMHMM2.0 (http://www.cbs.dtu.dk/services/TMHMM/) are underlined with black lines for SseG and gray lines for SseF. (B) The N terminus of SseF is exposed on the cytoplasmic face of the membrane. HeLa cells expressing N-terminal HA-tagged SseF were permeabilized with Triton X-100 (to permeabilize both plasma membrane and endomembranes) or digitonin (to selectively permeabilize the plasma membrane) and labeled with antibodies against the HA epitope and a luminal epitope of the Golgi protein TGN46. Bar, 5 µm. (C) Topology of SseF and SseG. The red stars indicate the point mutations. Download Figure S1, EPS file, 1.7 MB

Table S1  Primer sequences. Table S1, DOCX file, 0.01 MB
