# Supplementary material for: Salmonella Effectors SseF and SseG Interact with Mammalian Protein ACBD3 (GCP60) To Anchor Salmonella-Containing Vacuoles at the Golgi Network
Source: mBio. 2016 Jul 12;7(4):e00474-16. doi: 10.1128/mBio.00474-16 (PMC4958240; doi:10.1128/mBio.00474-16)
Supplement: Table S1 — Primer sequences. [file mbo004162904st1.docx]

Supplementary Table 1

| Name | Nucleotide sequence (5’ to 3’) |
| --- | --- |
| sseGyf | ATCGAATTCAAACCTGTTAGCCCAAATGC |
| sseGyr | ATCCTCGAGTTACTCCGGCGCACGTTG |
| sseFyf | ATCGAATTCAAAATTCATATTCCGTCAGCG |
| sseFyr | ATCCTCGAGTCATGGTTCTCCCCGAGATG |
| sseFn166yr | ATCCTCGAGTTA AATAGCGATTCCTGAACG |
| sseFc105yf | ATCGAATTC GGGGATGCTTGCTGTGCG |
| GBT641 | CTCGAGAAGACCTTGACATG |
| GBT1134r | GTATTGTTTGTGCACTTGCCG |
| sseGS67Gf | CCAAAAGAAATTTTTGGTTGGCAAACGGTTATTTTGGGC |
| sseGS67Gr | GCCCAAAATAACCGTTTGCCAACCAAAAATTTCTTTTGG |
| ACBD3_188_f | ATCGAATTCAAAAAAAGGAAGGAGGAAGAGGAGC |
| ACBD3_528_r | ATCCTCGAGTTATCTAGTATAATAGACTCTG |
| sseF1 | TCGATATCCAGAATGCGCAAATAATGGTTGATACTCTTATTGCTTAAA  TAACAGAACGAAATATGAAAATTCATATTCCGTCAGCGGCAAG |
| sseF2 | CCAACATAGAA ATAGCGATTCCTGAACGTATTAAT |
| sseF3 | ACGTTCAGGAATCGCTATTTCTATGTTGG |
| sseF4 | ATGAGCTCTCATGGTTCTCCCCGAGATG |
| sseF5 | CGGGATCCTCATGGTTCTCCCCGAGATG |
| sseG1 | CGGGATCCGATCATACATCTCGGGGAGAACCATGAAACCTG |
| sseGflagr | CAGAGCTCCTATTTATCGTCGTCATCTTTGTAGTCCTCCGGCG  CACGTTGTTCTGG |
